# The Effect of Single CpG Demethylation on the Pattern of DNA-Protein Binding

**DOI:** 10.3390/ijms20040914

**Published:** 2019-02-20

**Authors:** Barbara Sobiak, Wiesława Leśniak

**Affiliations:** Laboratory of Calcium Binding Proteins, Department of Molecular and Cellular Neurobiology, Nencki Institute of Experimental Biology of the Polish Academy of Sciences, 3 Pasteur Street, 02-093 Warsaw, Poland; b.sobiak@nencki.gov.pl

**Keywords:** cytosine methylation, CpG dinucleotide, epidermal differentiation complex, keratinocytes, PGLYRP3

## Abstract

Epidermal differentiation is a complex process and its regulation may involve epigenetic factors. Analysis of DNA methylation in 20 selected regions within the epidermal differentiation complex (EDC) gene cluster by targeted next-generation sequencing (NGS) detected no or only minor changes in methylation, mostly slight demethylation, occurring during the course of keratinocyte differentiation. However, a single CpG pair within the exon of the *PGLYRP3* gene underwent a pronounced demethylation concomitant with an increase in *PGLYRP3* expression. We have employed a DNA-affinity precipitation assay (DAPA) and mass spectrometry to examine changes in the composition of proteins that bind to DNA containing either methylated or unmethylated CpG. We found that the unmethylated probe attracted mostly RNA binding proteins, including splicing factors, which suggests that demethylation of this particular CpG may facilitate *PGLYRP3* transcription and/or pre-mRNA splicing.

## 1. Introduction

Keratinocyte differentiation is a highly coordinated process that ensures the proper function of the epidermis. In the course of differentiation, keratinocytes produce numerous specific proteins that serve to build a physical barrier against environmental hazards, infections and organismal water loss [1]. The epidermal differentiation complex (EDC) is a 1.7 Mb gene cluster on human chromosome 1q21 that houses many epidermis-specific genes [2]. Namely, EDC comprises genes of four protein families: the S100 proteins, the S100-fused type proteins (SFTP), the small proline-rich region (SPRR) proteins, late cornified envelope (LCE) proteins, and several other genes [3,4,5]. The EDC genes have a different onset of expression during fetal and postnatal development [6]. Furthermore, their expression changes during keratinocyte differentiation. The regulatory mechanisms that control EDC gene expression have not been elucidated yet.

DNA methylation is an epigenetic mechanism of gene expression regulation predominantly associated with gene silencing, as methylated cytosine residues attract components of chromatin repressive complexes [7]. DNA methylation pattern is acquired during early embryonic development and is characteristic for a given cell type [8]. Marked changes in the relatively stable methylation landscape are usually observed in cancer cells and each tumor type is associated with a unique set of methylated genes [9]. However, increasing evidence indicates that changes in cytosine methylation may occur in response to various environmental stimuli or diet [10], during cell or organismal aging [11], and during cell differentiation [12]. Involvement of DNA methylation in regulation of EDC gene expression upon keratinocyte differentiation may be considered based on studies showing differential expression of EDC genes upon treatment with DNMT inhibitor 5-azacytidine [13] or following DNMT1 knock-out [14]. Furthermore, changes in cytosine methylation in EDC genes occur in various skin pathologies, which are associated with disturbed epidermal differentiation [15]. We have recently compared the extent of DNA methylation in promoters and other regions of selected EDC genes in undifferentiated and differentiated primary human keratinocytes [16]. This analysis did not detect major changes in cytosine methylation that would correspond to the robust changes in expression of the examined genes. In the current analysis, using targeted next-generation sequencing (NGS), we have examined DNA methylation in 20 selected EDC areas located within DNase I hypersensitive sites and H3K27ac/H3K4me1 abundant sites, that is in presumptive regulatory regions, as a function of keratinocyte differentiation. We have detected marked demethylation of a single cytosine residue located in the 5th exon of the *PGLYRP3* gene, which associated with an increase in *PGLYRP3* mRNA level. Using a DNA-affinity precipitation assay (DAPA) and mass spectrometry we have investigated differences in the composition of proteins that bound to probes containing unmethylated or methylated CpG. This analysis revealed a marked switch in the set of bound proteins. In particular, significantly more RNA-binding proteins, including splicing factors, appeared to bind to the unmethylated probe suggesting that demethylation of a single cytosine residue may promote *PGLYRP3* transcription and/or pre-mRNA processing. 

## 2. Results

### 2.1. DNA Methylation Analysis

**Figure 1:**
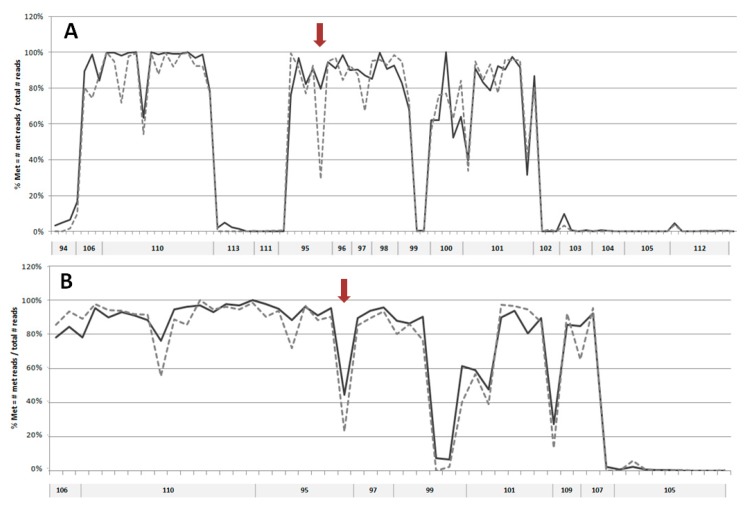
Targeted next-generation sequencing NGS analysis of CpG methylation in selected regions of EDC in DNA from undifferentiated and differentiated primary keratinocytes (NHEK). (**A**) Analysis performed on DNA from cells derived from an individual donor. (**B**) Analysis of highly methylated sequences shown in (A) in DNA from cells obtained from many donors. Sequences 109 and 107 could be read only in analysis B, and sequences 98 and 100 in analysis A. Solid line—undifferentiated keratinocytes; dashed line—differentiated keratinocytes. Arrows indicate the demethylated cytosine residue in sequence no. 95.

**Figure 2:**
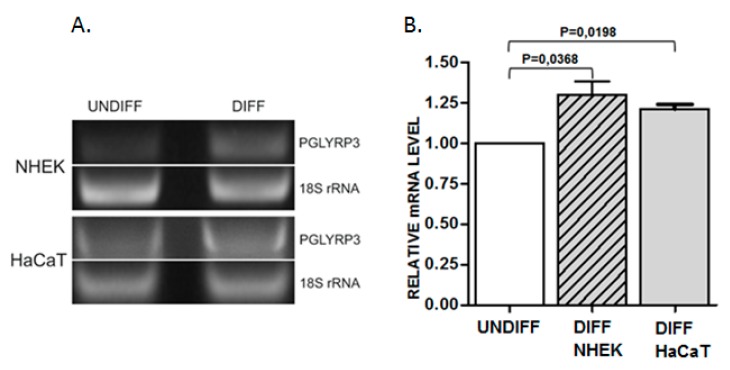
Analysis of *PGLYRP3* mRNA level in undifferentiated (undiff) and differentiated (diff) primary keratinocytes (NHEK) and HaCaT cells. (**A**) representative PCR results. (**B**) statistical analysis of results from 3 experiments. *PGLYRP3* mRNA level in undifferentiated NHEK or HaCaT cells is represented as 1.00 (white bar). The level in differentiated NHEK and HaCaT cells is represented by striped and gray bars, respectively. Data are presented as mean ± SEM.

In order to search for possible changes in DNA methylation that might occur during keratinocyte differentiation, we have selected 20 sequences mostly located within putative enhancer regions in EDC of normal human epidermal keratinocytes (NHEK), defined based on the presence of H3K27ac/H3K4m1 enriched and DNase I hypersensitive sites (USCS Genome Browser, GRCh37/hg19). Additional criteria for sequence selection were the preferable location in intergenic regions separating gene clusters or individual genes, number of CpGs, the density of transcription factor (TF) binding sites, and the presence of binding sites for methylation-sensitive TFs [17] and/or for TFs that bind to keratinocyte-specific enhancers [18]. The analyzed sequences covered 4781 bp and 103 CpG dinucleotides. The size and genomic location of these sequences together with the number of reads obtained in targeted NGS is presented in Table S1. The first analysis, performed on DNA from NHEK derived from an individual donor (Figure 1A), covered 17 sequences. It showed that, in general, the analyzed regions tend to be either highly methylated (75–100% methylation of individual CpGs) or unmethylated (0–10% methylation) with only one sequence (no. 99) showing a mixed methylation pattern. Upon differentiation, the level of methylation of individual CpGs remained unchanged or changed only slightly with most cytosine residues undergoing minor demethylation (by about 5%). There were only several cytosine residues that underwent marked demethylation. To verify these changes in cytosine methylation, the highly methylated regions were analyzed again in DNA derived from a heterogeneous keratinocyte population (NHEK obtained from many donors). The results (Figure 1B) confirmed that the overall methylation pattern of the studied regions is the same as in the material derived from an individual donor. The only cytosine residue that underwent marked demethylation according to both analyses was contained in sequence 95 and corresponded to the 5th exon of the *PGLYRP3* gene (Figure S1). To check whether this demethylation event coincided with altered gene expression, we have performed PCR analysis, which showed a concomitant increase in *PGLYRP3* mRNA level in differentiated versus undifferentiated NHEK and HaCaT cells (Figure 2).

### 2.2. Analysis of Protein Binding to a Sequence Containing the Variably Methylated CpG Pair 

**Figure 3:**
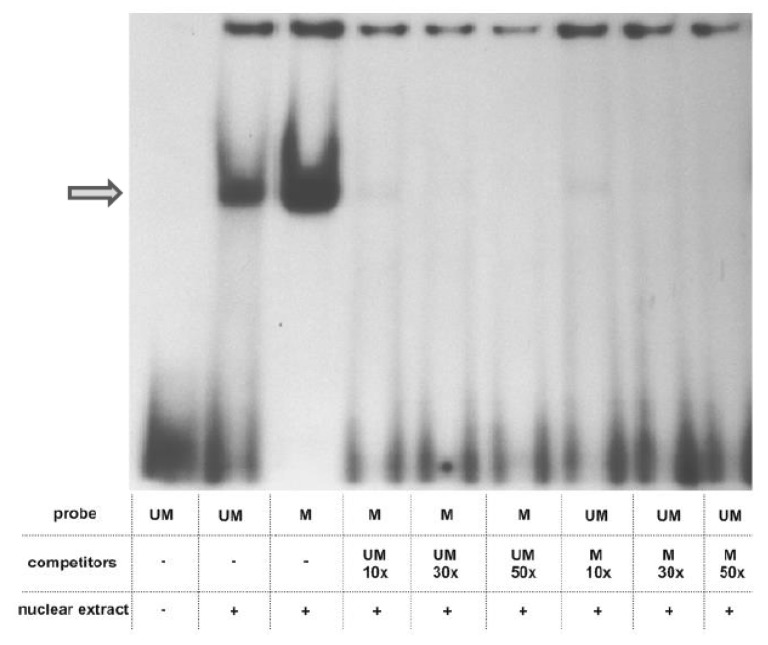
EMSA analysis. Nuclear extract of HaCaT cells (10 µg protein) was incubated with radioactively labelled probes containing the analyzed CpG in either an unmethylated or methylated form. Protein binding to each probe was competed with 10, 30 and 50 times excess of the other probe. The DNA-protein complex is indicated by an arrow.

**Figure 4:**
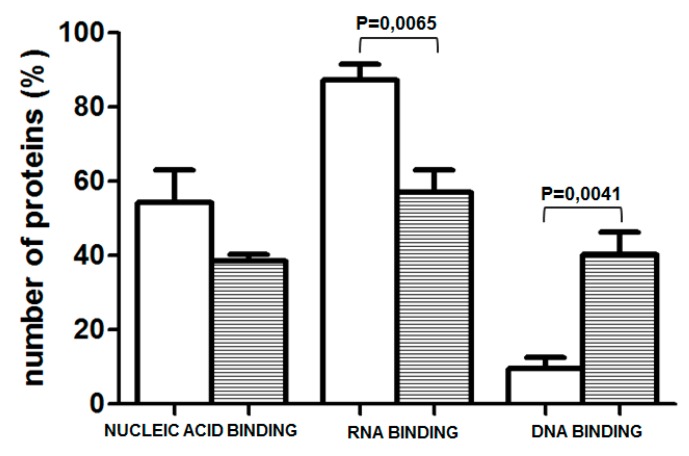
Percentage of proteins classified as nucleic acid binding and, within this category, as RNA- or DNA-binding proteins, bound to unmethylated (white bars) and methylated (striped bars). Data obtained in 4 experiments are presented as mean ± SEM.

To explore whether the prominent change in methylation of one of the examined CpGs, which occurred during differentiation of NHEK, induces a switch in the amount/composition of protein-DNA complexes formed in its vicinity, we have performed EMSA assays using a 26 bp long oligonucleotide containing the examined CpG pair in either unmethylated or methylated form. As shown in Figure 3, prominent protein complexes of identical mobility bound to both probes. The same mobility of the complexes and the fact that the binding of each complex could be competed by the excess of both the unmethylated and methylated probes suggested that their binding, although specific, was not critically dependent on the CpG methylation state. Therefore, to look for differently bound proteins, we have performed a DNA-affinity precipitation assay (DAPA) followed by mass spectrometry analysis. To focus our analysis on proteins whose binding was dependent on the presence of the CpG dinucleotide in the examined sequence, we have preincubated the nuclear extract from HaCaT cells with a mutated sequence, containing TA instead of CG. The unbound fraction was then incubated with either the unmethylated or methylated sequence. Altogether, four experiments were performed. Among all the bound proteins, we searched for those that bound uniquely to either of the two sequences. Depending on the experiment, we identified 44–149 proteins that bound only to the unmethylated sequence and 7–144 that bound to the methylated probe. As could be expected, proteins classified by the PANTHER program as nucleic acid binding proteins and transcription factors constituted the largest class of proteins bound to each sequence. Namely, those proteins accounted for 54.27% of all proteins bound to the unmethylated sequence and for 38.57% of proteins bound to the methylated one (Figure 4). As to the unmethylated probe, 10–37% of these proteins, depending on the experiment, were identified in at least two analyses (and were not identified as bound to the methylated probe in any of the analyses). For the methylated probe, the set of bound proteins was more heterogeneous across experiments and the respective percentage amounted to 11–28%. Most interestingly, when nucleic acid binding proteins were further classified into DNA- and RNA-binding proteins, the proportion was definitely different for the two sequences (Figure 4). RNA-binding proteins constituted, on average, 87.25% of proteins bound to the unmethylated sequence, that is significantly more than in the case of the methylated one (57.15%). Of those, splicing factors of the SRSF family, RPLs or SMARC proteins were found, rather consistently, among proteins bound to the unmethylated probe (Table S2). On the other hand, there were, on average, more proteins belonging to the DNA-binding protein category bound to the methylated probe (Figure 4). Of note, MBD2, a protein that binds methylated cytosine, was identified in two analyses. 

## 3. Discussion

The targeted NGS analysis of 20 selected sequences within EDC localized in presumptive regulatory/enhancer regions revealed that, in these particular regions, DNA tends to be either highly (75–100%) or hardly (0–5%) methylated, concordant with the general pattern of genome methylation. Of note, as indicated in the Methods Section, bisulfite-based techniques cannot distinguish between methylated and hydroxymethylated cytosine so the presence of the latter modification in the examined regions cannot be excluded. Upon differentiation of primary human keratinocytes, the majority of the analyzed CpG residues were subject to only minor changes in the extent of methylation—with slight demethylation (1–5%) representing the prevailing trend. As an exception to this rule, we noticed a prominent decrease in methylation of a single CpG dinucleotide in primary keratinocytes derived both from an individual donor and in a keratinocyte population derived from many donors. The consistency and uniqueness of this demethylation event prompted us to examine it further. 

The CpG pair undergoing demethylation is located in the 5th exon of the *PGLYRP3* gene (Figure S1). The peptidoglycan recognition protein 3 (PGLYRP3) encoded by this gene is an innate immunity molecule, one of the four mammalian representatives of the PGRP protein family [19]. Two *PGLYRP* genes, encoding PGLYRP3 and PGLYRP4, are located in EDC. According to an interesting hypothesis, the ancestral *PGRYLP* and *S100* genes, through multiple fusion and duplication events, could have given rise to all EDC gene families [20]. PGLYRP3 and PGLYRP4 are expressed mainly in the epidermis and epidermal appendages such as hair follicles, sebaceous glands and sweat glands [19]. Both proteins can be secreted and are bactericidal for many pathogenic and nonpathogenic bacteria. Changes in PGLYRP3 expression have been described in the context of microbial components and Toll-like receptor activation [21] or in skin pathologies [22], but less information is available concerning PGLYRP3 expression during epidermal differentiation. Our results indicate that the level of *PGLYRP3* mRNA increased during differentiation of both primary human keratinocytes (NHEK) and keratinocyte-derived HaCaT cells. This increase occurs concomitantly with the loss of methylation of the CpG dinucleotide in the 5th exon of this gene, but a causative link between these two events could not be proved at the moment. 

There are numerous reports that methylation of a single CpG pair, either in gene promoters [23] or introns and exons [24] can silence gene expression. Conversely, demethylation at single CpG residues should be able to trigger gene expression and such events have been observed in gene promoters [25,26]. It is not known how methylation or demethylation of a single CpG residue, especially within an otherwise unmethylated or methylated genomic area, is able to influence the structure of chromatin and DNA-protein interactions to execute a profound change in transcription rate. To get some insight into this issue, we have performed EMSA using double-stranded oligonucleotides, which matched the sequence containing the examined CpG pair, in either methylated or unmethylated form. The analysis detected a specific protein complex, which, however, bound to the examined sequence regardless of its methylation and could not, therefore, account for an altered expression rate. To further pursue this issue we employed DNA-affinity precipitation assay (DAPA) followed by mass spectrometry analysis to detect proteins that bound differentially to the methylated or unmethylated probes. This assay, followed by Western blot analysis, is routinely used to examine the binding of transcription factors and other proteins of interest to the analyzed sequence [27]. The combination of DAPA and mass spectrometry has been used earlier, for example, to compare protein binding to hydroxymethylcytosine and formylcytosine [28]. Using this experimental setup, we were able to discern a set of proteins that bound preferably to each probe. Interestingly, the unmethylated probe attracted mostly RNA binding proteins, including splicing factors, which were poorly represented or absent from the pool of proteins bound to the methylated probe. Given that the cytosine residue undergoing prominent demethylation during keratinocyte differentiation is located within an exon, this may indicate that the unmethylated sequence is more accessible to the transcriptional and/or spliceosomal machinery than the methylated one. Thus, loss of methylation could result in increased *PGLYRP3* expression upon differentiation. DNA methylation in exons is implicated in both transcription and splicing. Methylation within exons is generally believed to favor transcription [12]. On the other hand, the exact role of cytosine methylation in the regulation of alternative splicing is still a subject of intensive studies (Maor et al., 2015) [29]. Our observation that splicing factors of the SRSF and U2AF family bind preferably to unmethylated DNA sequence may be a valuable contribution to solving this issue. 

## 4. Methods

### 4.1. Cell Culture

Normal human epidermal keratinocytes (NHEK) were purchased from PromoCell and used at early passages. Cells were maintained in Keratinocyte Growth Medium 2 (PromoCell, Heidelberg, Germany) supplemented with 0.06 mM calcium chloride, human keratinocyte growth supplement (HKGS), 100 U/mL penicillin and 100 µg/mL streptomycin. Cell differentiation was induced by increasing calcium chloride concentration to 1.8 mM. HaCaT cells, spontaneously immortalized human keratinocytes, purchased from Cell line Service (Germany), were cultured and differentiated as described in Reference [30]. Cells were cultured in a cell incubator at 37 °C and 5% CO_2_.

### 4.2. DNA Methylation Analysis

DNA was isolated from undifferentiated and differentiated NHEK using a standard method and conditions as described in Sobiak et al., 2016. The DNA was then subjected to bisulfite treatment, which converts cytosine into uracil while methylated (or hydroxymethylated) cytosine remains intact. Twenty selected regions within EDC, 119–314 bp in length, were amplified and subjected to targeted next-generation sequencing (NGS) reaction performed by EpigenDX (Hopkinton, MA, USA). The minimal sequencing depth was set at 30. The analysis was performed twice: once on DNA from NHEK derived from an individual donor and the second time on DNA from cell population obtained from many donors. 

### 4.3. Preparation of Nuclear Extracts

HaCaT cells grown in a 10 cm plate were scraped in PBS and pelleted by centrifugation at 600× *g*. Cells were then resuspended in 0.5 mL of hypotonic buffer (20 mM Hepes pH 8.0, 0.2% Igepal CA-630, 1 mM EDTA, 1 mM DTT) supplemented with protease inhibitor cocktail (Roche) and incubated for 10 min on ice. The lysate was centrifuged at 5000× *g* for 10 min and the resulting pellet containing cell nuclei was incubated for 30 min on ice in the extraction buffer (20 mM Hepes pH 8.0, 450 mM NaCl, 20% glycerol, 1 mM EDTA, 1 mM DTT and protease inhibitor cocktail) with occasional mixing. Following centrifugation at 12,000× *g* for 15 min at 4 °C the supernatant was collected and, after protein determination with protein assay dye reagent (Bio-Rad, Hercules, CA, USA), used immediately or portioned and kept at −20 °C.

### 4.4. RNA Isolation and PCR

Total RNA was isolated from undifferentiated and differentiated NHEK and HaCaT cells using RNeasy mini kit (Qiagen, Hilden, Germany) according to the manufacturer’s instructions. Two microgram of RNA was used for cDNA synthesis catalyzed by M-MLV reverse transcriptase (Sigma Aldrich, St. Louis, MO, USA) in the presence of random nonamers (Sigma Aldrich). To assess the level of *PGLYRP3* transcript, a PCR reaction was conducted with the following primers: 5′-CGCCCAGGTATATTCAGCCA-3′ and 5′-CCGTGTGTCCATGTGAAAGG-3′ for 35 cycles.

### 4.5. Electrophoretic Mobility Shift Assay (EMSA)

A 26 base long oligonucleotide, the sequence of which corresponded to the fragment of the probe no. 95 containing the differentially methylated cytosine residue, was synthesized at the DNA Sequencing and Oligonucleotides Synthesis Laboratory, Institute of Biochemistry and Biophysics, PAS, Warsaw, Poland, in a form containing either unmethylated (5′-TCCCAAGCAGATCGTTTGATGATGTT-3’) or methylated cytosine (5′-TCCCAAGCAGATmCGTTTGATGATGTT-3’). Both oligonucleotides were hybridized with complementary strands and radioactively labelled using T4 kinase and (γ^32^P) ATP. An amount of probe equivalent to 100,000 cpm was incubated with 10 µg of nuclear extract from HaCaT cells (grown in standard DMEM). The labeling and binding reactions and the subsequent gel electrophoresis were performed essentially as described earlier [31]. 

### 4.6. DNA Affinity Precipitation Assay (DAPA)

The two oligonucleotides used in EMSA (5′-TCCCAAGCAGATCGTTTGATGATGTT-3’ and 5′ -TCCCAAGCAGATmCGTTTGATGATGTT-3’) and a corresponding oligonucleotide with the CG pair mutated (5′-TCCCAAGCAGATATTTTGATGATGTT-3’) were synthesized and biotinylated at the 5′-termini by the same facility as oligonucleotides used for EMSA. They were then annealed with their complementary, non-biotinylated, strands. The DNA affinity precipitation assay was performed as follows: 600 μg of nuclear extract diluted 5 times in binding buffer (2 µg herring sperm DNA, 20 mM HEPES, pH 7.9, 2 mM MgCl_2_, 0.2 mM EDTA, 1 mM DTT, and 10% (*v*/*v*) glycerol) was first precleared by incubation with 10 μL of streptavidin-agarose beads for 1 h. After separation of the beads, the supernatant was first incubated with 2 μg of the mutated DNA probe and 40 μL of streptavidin-agarose for 1 h at 4 °C. The unbound fraction was divided into two equal portions, which were incubated with either the methylated or unmethylated probe as above. Beads were collected and washed three times with the binding buffer containing 0.5% Nonidet P-40. Proteins bound to the beads were eluted with elution buffer (5 mM Tris-HCl, pH 7.5, 0.5 mM EDTA, 0.5 M NaCl), and subjected to mass spectrometry analysis performed at the Mass Spectrometry Laboratory, Institute of Biochemistry and Biophysics, PAS, Warsaw, on an Orbitrap spectrometer. Data were analyzed by the Mascot program (www.matrixscience.com) based on the NCBI sequence database. The cut-off point was set at Mascot score 50. 

### 4.7. Statistical Analysis

Analysis of proteins retrieved by Mascot was performed using PANTHER (protein analysis through evolutionary relationships) (http://www.pantherdb.org) and Fisher’s exact test with FDR correction. Statistical data analysis of data presented in Figure 2 and Figure 4 was performed using Student’s *t*-test.

## 5. Conclusions

Demethylation of a single CpG in the 5th exon of *PGLYRP3* facilitates binding of proteins involved in RNA processing, including splicing, and may contribute to the enhanced expression of this protein during keratinocyte differentiation. 

## Supplementary Materials

Supplementary materials can be found at http://www.mdpi.com/1422-0067/20/4/914/s1.
