# Supplementary material for: The Effect of Single CpG Demethylation on the Pattern of DNA-Protein Binding
Source: Int J Mol Sci. 2019 Feb 20;20(4):914. doi: 10.3390/ijms20040914 (PMC6413078; doi:10.3390/ijms20040914)
Supplement: Supplementary file 1 [file ijms-20-00914-s001.zip › Table S1.pdf]

Table S1. Genomic location of EDC regions examined by targeted NGS.

Sequencing depth	Assay ID	Amplicon Location (GCRh37/hg19)	size
>30 x	ADS5**094**	Chr1:152163957-152164130	172
>30 x	ADS5**106**	Chr1:152555752-152555979	228
>30 x	ADS5**110**	Chr1:152732780-152733093	314
>30 x	ADS5**113**	Chr1:152926868-152927147	280
>30 x	ADS5**111**	Chr1:152939777-152940043	267
>30 x	ADS5**095**	Chr1:153274856-153275087	236
>30 x	ADS5**096**	Chr1:153277360-153277478	119
>30 x	ADS5**097**	Chr1:153312802-153313011	210
>30 x	ADS5**098**	Chr1:153320207-153320461	255
>30 x	ADS5**099**	Chr1:153468125-153468417	293
>30 x	ADS5**100**	Chr1:153520072-153520343	272
>30 x	ADS5**101**	Chr1:153520796-153521007	213
>30 x	ADS5**102**	Chr1:153521814-153522061	248
>30 x	ADS5**103**	Chr1:153538200-153538487	288
<10 x	ADS5**104**	Chr1:153538996-153539286	291
>30x	ADS5**105**	Chr1:153540197-153540467	271
>30 x	ADS5**112**	Chr1:153585466-153585611	146
>30 x	ADS5**109**	Chr1:152973332-152973504	173
>30 x	ADS5**107**	Chr1:152552991-152553285	228
failed	ADS5**108**	Chr1:152880966-152881242	277
