# Supplementary material for: The Effect of Single CpG Demethylation on the Pattern of DNA-Protein Binding
Source: Int J Mol Sci. 2019 Feb 20;20(4):914. doi: 10.3390/ijms20040914 (PMC6413078; doi:10.3390/ijms20040914)
Supplement: Supplementary file 1 [file ijms-20-00914-s001.zip › Table S2.pdf]

Table S2. List of proteins bound selectively to the unmethylated and methylated probes in four DAPA experiments.

EXP 1		EXP 2		EXP 3		EXP 4	
unmethylated	methylated	unmethylated	methylated	unmethylated	methylated	unmethylated	methylated
HIST1H1D	RBBP7	DDX39B	TMPO	ACTC1	CTNNA2	MCM4	DES
EEF1A2	DDX5	DEK	PLEC	LMNB2	TUBB6	RSBN1L	ACADVL
ACTA2	MBD2	RPS2	EWSR1	UQCRC1	ACSL4	HDGFL2	HNRNPA3
ANXA2	ANXA2P2	U2AF2	DDX39A	G3BP2	SLC25A4	POLR1C	ATP1A1
ATF1	DSG1	EPPK1	DCD	AP1B1	INTS3	KDM2A	LRRC47
PTBP1	TMPO	RPS6	SEC31B	SMARCC1	DHX9	CAPRIN1	ACSL4
RPLP0	TCOF1	RPS9	RCC1	KDM2A	C14orf166	UBTF	ME2
SOX15	LRRC59	PHB		BCLAF1	LEMD3	RANBP1	MTA2
MAP4	ACADVL	CPSF6		SEPT2	RBBP4	LMNA	SLC1A3
EIF2S1	ITGA6	SFPQ		HCFC1	ATP2A2	MAX	DDRGK1
RECQL	RPS6	SLC25A5		SRSF2	FDFT1	RPS20	HMGN4
SRSF7	ALKBH5	H2AFZ		NAT10	CTTN	ACTN4	SEC61B
SPTBN1	FAU	RPS7		PTRH2	UGT1A6	U2AF2	PHB
MRPL12	NACA	CKAP4		TOR1AIP2	TAP1	PRSS3	CD3EAP
SRSF8	SRSF2	RPLP0		NDUFAB1	NDUFAF1	PRSS1	PRSS2
RPL27	FLG	HMGN2		DSG1	DNAJA1	NASP	DTD1
S100A7A	SNRPD3	MATR3		CSTF2	TBRG4	POU2F1	RBBP7
SRSF6	CCDC40	RPS13		TGM3	MT	PRPF19	RPN1
HNRNPAB	KCNAB1	TRIM28		POLR2A	XPO1	HIST2H2BF	TP63
SPTAN1	CALML5	RPL27A		MAPRE1	RETSAT	POLD3	IMMT
EPPK1	LAD1	TMPRSS13		RPL10	MTA1	HRNR	SUPT6H
PLEC	LETM1	RPS10		ITGB1	PAF1	AGRN	LRPPRC
SYNCRIP	TADA2B	SEC31A		STAG2	SCCPDH	P4HB	ACTN1
RPS2	TFAM	CYFIP2		ATP2A1	ATP2B1	C1QBP	PNKP
BCAP31	AGPAT1	SRSF9		MRPL53	RPS2	SRSF10	ATP2A2
FTCDNL1	KTN1	HNRNPH1		CSTF3	MBOAT7	DDX3X	RPL27A
IGHA1	ATP5EP2	MT-CO2		NCAPH	HADH	SHMT2	MIA3
SLC25A5	HIST1H2AG	SNRPD1		SEPT7	NCLN	MARCKS	PSMC1
RPL31	ORC2	RPS4X		KIF5B	KIF4A	ANP32A	BCAP31
RPL34	KIF4A	CCDC25		RBM3	RPS18	LIG1	PAF1
EBNA1BP2	THEM6	RPL7		RPP30	MBD2	FLNA	MT-CO2
G3BP2	RBM3	RPL26L1		PRMT5	ZNF207	FLNB	DHX15
RPL22	USP39	KATNAL2		MRPL55	IKBIP	RPL12	SSR4
ACOT9	GTF2F2	RPL18		LAMC1	CLPX	SRSF7	POLR3A
CANX	HDAC1	RPL12		PPP1R7	NDC1	DDX17	KIF4B
TCERG1	LSM3	CALML5		COPG1	TMED10	TFAP4	H3F3C
RPS12	DHCR7	SERBP1		P4HA2	ORC2	AIMP1	MORF4L2
VRK1	EFCAB14	RPS17		SMARCA5	LPL	DBT	POLR3F
FOXK1	TRIM28	RPL35		TFG	PBXIP1	LSM3	KPRP
FAM223A	ILF3	SNRPF		UBTF	EDF1	RPL13	XP32
ABCA12	DDOST	RPL31		RCN2	GCN1	ACIN1	FOXK1
UBB	HRNR	RPLP1		DTX3L	TMEM109	MRPL40	SLC25A31
FAM98B		RPL6		FUBP3	FARSA	ITGB4	NONO
RPL35A		NUDT21		LAD1	ANO1	RPL23A	MGME1

LRMP	HNRNPUL2	STK25	SF3B4	TMPRSS11F	OGDH
	RPL13AP3	MRPL2	MRPL14	DDX18	S100A14
	RPL29	WDR18	SLC12A2	DNAJC9	
		PNN	KIAA0391	S100A9	
		PSMA3	FOXK1	MRPS7	
		STT3B	PPP1CC	RPS12	
		LAMB3	HYPK	STIP1	
		ARCN1	PE	GTF2F1	
		XAB2	TOR1AIP1	SMC4	
		SOX7	DRAP1	KIF22	
		MRPS7	SPTLC3	RPS16	
		SRSF3	RPS20	BASP1	
		RPS15A	BUD31	PLCB3	
		MYO18A	SPCS2	HNRNPAB	
		TFB1M	BRD3	TRIM25	
		RPL24	RBM4	RPS2	
		RPL28	RPS25	RPS6	
		LSM14B	EIF3CL	MATR3	
		CSNK2A2	SCD	FAM47DP	
		ACLY	PDCD10	API5	
		RNF213	MORF4L1	RPL7A	
		SCRIB	MORF4L2	C19orf53	
		LRRC1	SLC25A11	DARS2	
		PRPF3	EXOC1	SNRPD1	
		PSMD6	ALG9	XRN2	
		SMARCB1	MLH1		
		COLGALT1	CHP1		
		KHSRP	RDH13		
		TECR	HELLS		
		SPTLC2	FAF2		
		ZRANB2	WDHD1		
		USP28	NR3C1		
		MARS	FANCI		
		LARS	POLR2E		
		DDX47	CAVIN3		
		DDX24	ZC3H11A		
		FKBP4	TPT1		
		UHRF2	GTF3C4		
		NUP50	KIF22		
		AP2M1	CYBA		
		BAG6	CRNKL1		
		ARG1	PBX2		
		MRPL28	HLA		
		NOP9	HLA		
		SNU13	EIF2AK2		
		AGRN	ATP5J		
		AKAP8	SRSF6		
		PSMD8	EMD		
		ATRX	MRPL21		
		EXOSC10	DENR		

		CLPP	RNASEH2B
		UPF1	GALNT3
		LLGL1	NME1
		NHP2	LPGAT1
		DDX50	DUOX1
		NFYA	LRWD1
		METTL15	MRPL11
		SSR3	DSG3
		KPNA5	NAP1L4
		RAD23B	FECH
		PSMA4	ESYT2
		EPN1	GNAS
		PSMB6	ITGA3
		MRPL32	LRRC41
		MRPL24	OAS2
		NUP133	MRPL50
		EIF5	CNPY2
		PPP2CB	PCDH1
		SRBD1	LUC7L2
		RNF40	LUC7L
		LYAR	TOMM20
		APLP2	FKBP11
		HOMEZ	RPA3
		GLYR1	LIMA1
		VASP	ATP5J2
		IFIT5	FAR1
		PPP1CB	ALDH18A1
		WDR82	TIMM50
		SMAP	TSN
		AIMP1	GALNT2
		FOXRED1	NUSAP1
		RNPS1	UQCC2
		RBM10	XPA
		GTF3C1	RCOR1
		TSNAX	POGLUT1
		MAD1L1	DHRS7
		STX5	NDUFA9
		CCNK	THUMPD1
		TERF2IP	MRGBP
		RBM27	TMEM205
		KIF11	CORO1B
		EXOC2	CLPB
		PKP1	ELOB
		PBRM1	SSBP1
		MRPS14	BZW2
		RXRA	PRSS3
		PABPN1	MRPL17
		MRPL38	SEC11A
		FIP1L1	FADS1
		NIPSNAP1	TP63

		BCKDHB	SLC25A24
		ERMP1	PSMB2
		RANGAP1	
		MRPL16	
		DIDO1	
		GPD2	
		VIRMA	
		BRD4	
		EXOSC2	
		MRPL58	
