# Supplementary figures and images for: The Effect of Single CpG Demethylation on the Pattern of DNA-Protein Binding
Source: Int J Mol Sci. 2019 Feb 20;20(4):914. doi: 10.3390/ijms20040914 (PMC6413078; doi:10.3390/ijms20040914)

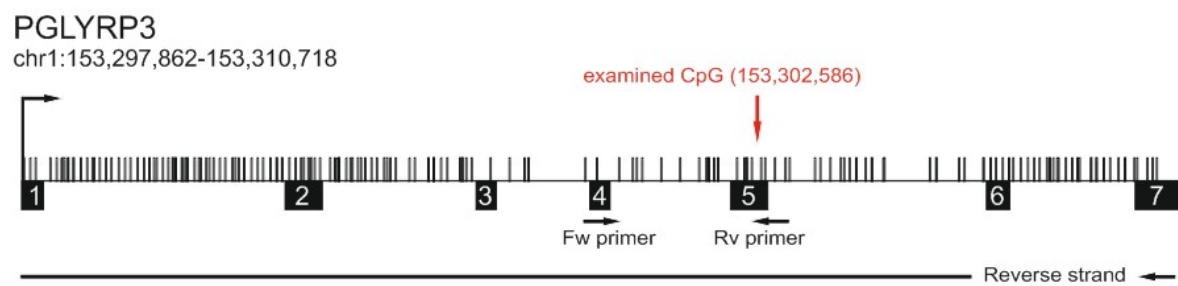

**Figure S1.** Schematic representation of *PGLYRP3*.

Supplement: Supplementary file 1 [file ijms-20-00914-s001.zip › Figure S1.pdf]
